# Nephrotoxicity of Anti-Angiogenic Therapies

**DOI:** 10.3390/diagnostics11040640

**Published:** 2021-04-01

**Authors:** Margaux Van Wynsberghe, Joanne Flejeo, Hamza Sakhi, Mario Ollero, Dil Sahali, Hassan Izzedine, Carole Henique

**Affiliations:** 1 INSERM, Institut Mondor de Recherche Biomédicale, Paris Est Creteil University, F-94010 Creteil, France; margaux.van-wynsberghe@inserm.fr (M.V.W.); joanne.flejeo@gmail.com (J.F.); hamza.sakhi@inserm.fr (H.S.); mario.ollero@inserm.fr (M.O.); dil.sahali@inserm.fr (D.S.); 2 Service de Néphrologie, CHU Pontchaillou, F-35000 Rennes, France; 3 Service de Néphrologie, AP-HP, Groupe Henri-Mondor Albert-Chenevier, F-94010 Creteil, France; 4 Department of Nephrology, Peupliers Private Hospital, F-75013 Paris, France; hassan.izzedine@aphp.fr

**Keywords:** kidney, VEGF signaling, toxicity

## Abstract

The use of inhibitors of vascular endothelial growth factor (VEGF)/vascular endothelial growth factor receptor 2 (VEGFR2) signaling for the treatment of cancer has increased over the last decade. This signaling pathway plays a fundamental role in angiogenesis and also in kidney physiology. The emergence of anti-angiogenic therapies has led to adverse nephrotoxic effects, despite improving the outcomes of patients. In this review, we will present the different anti-angiogenic therapies targeting the VEGFR pathway in association with the incidence of renal manifestations during their use. In addition, we will discuss, in detail, the pathophysiological mechanisms of frequent renal diseases such as hypertension, proteinuria, renal dysfunction, and electrolyte disorders. Finally, we will outline the cellular damage described following these therapies.

## 1. Introduction

In a normal adult, most vasculature is quiescent, with only 0.01% of endothelial cells undergoing division. In various diseases, for example, pre-eclampsia, diabetic retinopathy, and hemangioma, inflammation and hypoxia may contribute to abnormal angiogenesis [1]. Pathological angiogenesis is one of the cancer hallmarks and is defined as the development of new tumor blood vessels from the pre-existing ones. There are several molecular players involved in these mechanisms, but the major mediator of tumor angiogenesis is the vascular endothelial growth factor (VEGF) produced by tumor cells. It signals through a VEGF receptor with tyrosine kinase activity that stimulates endothelial cells to proliferate and migrate. This pathway is an established therapeutic target in many cancers [2]. Indeed, angiogenesis inhibitors targeting the VEGF ligand (anti-VEGF) or inhibiting its receptors (tyrosine kinase inhibitors, TKIs) are widely used in oncology.

Many of these agents improve the outcomes of patients by extending overall survival and/or progression-free survival, but their safety profiles remain unclear and they might lead to previously unknown or poorly recognized adverse events, particularly nephrotoxicity, reported in 10–20% of patients [3,4,5]. TKIs are selective for one or several specific targets but are not selective regarding different cell types or tissues. The mechanisms that link anti-cancer therapy by TKI or anti-VEGF to glomerular cell dysfunction, hypertension, and nephrotic proteinuria are the subject of intense basic and translational research [6,7].

This review provides an overview on the nephrotoxicity of anti-angiogenic therapies.

## 2. VEGF, Pro-Angiogenic Factor, Renal Expression

The most important molecule that promotes angiogenesis and increases vascular permeability is VEGF-A (also called VEGF). It is a member of the mammalian platelet-derived growth factor (PDGF) supergene family which also includes VEGF-B, VEGF-C, VEGF-D and placental growth factor (PlGF). VEGF signals mainly through the VEGF receptor 2 (VEGFR2) and also binds VEGFR1, both members of the VEGFR tyrosine kinase family (VEGFR1, VEGFR2, and VEGFR3, encoded by the genes *FLT1 (Fms Related Receptor Tyrosine Kinase 1), KDR (Kinase Insert Domain Receptor)*, and *FLT4 (Fms Related Receptor Tyrosine Kinase 4)*, respectively), which is expressed at elevated levels in endothelial cells [8,9]. VEGFR1 has a substantially higher affinity for VEGF than for VEGFR2 but mediates a much lower pro-angiogenic activity [10]. VEGFR3 is mainly restricted to lymphatic endothelial cells.

Receptor tyrosine kinases (RTKs) have a similar molecular structure, with a ligand-binding site in the extracellular domain, a transmembrane region, and a cytoplasmic region that contains the protein tyrosine kinase (TK) domain with an ATP-binding site. Growth factors bind their specific extracellular domain and activate RTKs by inducing receptor dimerization, which, in turn, activates their autophosphorylation, thus initiating a cascade of downstream signaling events [11]. They are key regulators of critical cellular processes such as proliferation and differentiation, cell survival, metabolism, and migration.

Several studies have documented the importance of VEGF signaling in maintaining glomerular integrity [12,13,14]. In the glomerulus, VEGF is expressed and secreted by podocytes, which are highly differentiated visceral epithelial cells with foot processes, located on the urinary side of the glomerular basement membrane. VEGFRs are expressed on the surface of both endothelial cells and podocytes, even if the latter remains controversial [15,16,17,18,19]. Indeed, the autocrine effect of VEGF on the podocyte has been discussed. Bertuccio and Veron et al. have established that VEGFR2 is expressed in adult mouse podocytes and glomerular endothelial cells [17,20]. Müller-Deil et al. have identified VEGFRs in murine and human podocyte cell cultures [18], while Wang et al. have shown the expression and distribution of VEGFR2 both in endothelial cells and in podocytes by immune electron microscopy and immunofluorescence in human patients [16]. However, when Eremina et al. induced a podocyte-specific deletion of VEGFR2 in mice, they did not observe any effect on either glomerular development or function. They failed to detect any expression of VEGFR2 in podocytes [15].

This configuration allows a VEGF-mediated epithelial–endothelial crosstalk and contributes to the functional glomerular filtration barrier through survival, proliferation, and/or differentiation of the adjacent fenestrated glomerular capillary endothelial cells [21]. VEGF is known to exert bidirectional effects on podocytes, depending on its expression level. In adult mice, chronic VEGF knockout induced thrombotic microangiopathy (TMA) [13]. The best documented example for a pathological role of VEGF inhibition in the kidney is pre-eclampsia. Pre-eclampsia is a hypertensive disorder peculiar to pregnancy (4–5% of pregnant women). This systemic syndrome appears to originate in the placenta and is characterized by widespread maternal endothelial dysfunction, the presence of new-onset hypertension, and proteinuria or other end-organ damage occurring after 20 weeks of gestation [22]. The pathogenesis of pre-eclampsia relies on placental ischemia, abnormal spiral artery remodeling, and oxidative stress, leading to increased systemic levels of the circulating soluble form of VEGFR1 (also known as sFlt1, soluble Fms-like tyrosine kinase-1), an antagonist of VEGF, and PlGF [23,24]. Indeed, excess levels of the anti-angiogenic factor sFlt1 are associated with decreased circulating levels of VEGF and PlGF, resulting in maternal endothelial dysfunction, glomerular endotheliosis, and proteinuria, which may progress to thrombotic microangiopathy [22,25,26]. By contrast, podocyte VEGF overexpression induces collapsing glomerulopathy [17,21]. Renal expression of VEGF and its receptors is upregulated in patients with diabetic nephropathy, which induces new vessel formation in the kidney, stimulates renal hypertrophy, and causes proteinuria in experimental models [27,28]. An in vitro study showed that VEGF promotes podocyte survival through an autocrine pathway involving VEGFR2, inducing podocin upregulation and its association with CD2AP (CD2-associated protein), an adaptor molecule regulating podocyte actin polymerization [29]. Moreover, VEGFR2 interacts with nephrin, an adhesion protein and key regulator of podocyte survival via Akt signaling. Indeed, VEGFR2 is rapidly phosphorylated in response to VEGF and recruits the Src kinase Fyn, which binds to nephrin and initiates a cascade of phosphorylation, leading to actin cytoskeleton polymerization and actin stress fiber formation [20,30].

## 3. Anti-Angiogenic Drugs

Solid tumor growth and metastasis spreading depend on angiogenesis. Various signals may trigger the angiogenic switch, for example, metabolic stress (hypoxia, low pH, or hypoglycemia), mechanical stress, immune/inflammatory response, and genetic mutations [1,31,32]. VEGF secreted by tumor cells stimulates endothelial cell proliferation and survival, leading to the establishment of new blood vessels [10]. Indeed, hypoxia regulates angiogenesis at every step of this process through multiple pathways, including VEGF. The master oxygen homeostasis regulators of this process are the hypoxia-inducing factors, HIFs. The founding member of this family is HIF-1α [33].

In 1971, Folkman introduced anti-angiogenesis as a new anti-cancer strategy [34]. However, only in 2004, the US Food and Drug Administration (FDA) approved bevacizumab, a humanized anti-VEGF monoclonal antibody, for metastatic colorectal cancer treatment [35]. To date, several anti-angiogenic therapies have been developed and approved to treat cancers and other activated VEGFR-related diseases.

### 3.1. Anti-VEGF mAb and Tyrosine Kinase Inhibitors

**Figure 1:**
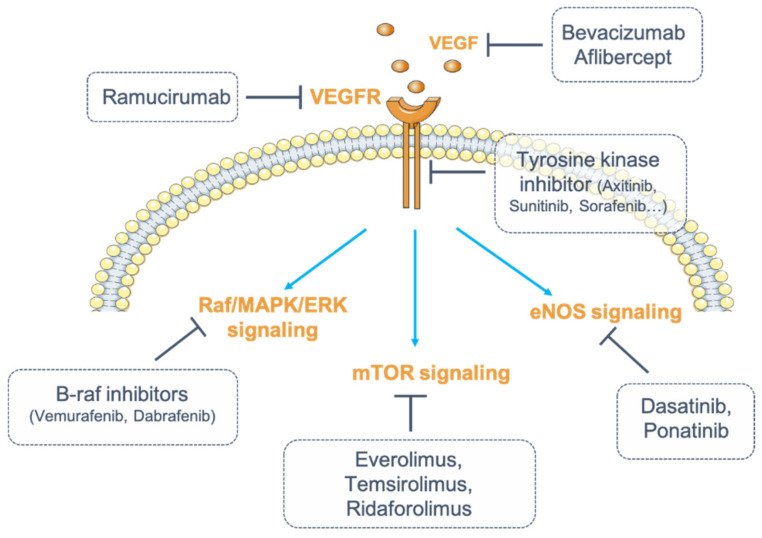
Inhibition of vascular endothelial growth factor (VEGF)/vascular endothelial growth factor receptor (VEGFR) signaling. Numerous strategies exist to inhibit VEGF/VEGFR signaling. VEGF can be blocked by monoclonal antibodies (mAbs) (bevacizumab) or by fusion proteins (aflibercept). Its receptor, VEGFR, can be targeted by fully humanized monoclonal antibodies (ramucirumab). The receptor can also be targeted for its intracellular tyrosine kinase activity (tyrosine kinase inhibitors, TKIs). Finally, one strategy consists of inhibiting the downstream signaling pathways of VEGFR by targeting either the Raf (Rapidly Accelerated Fibrosarcoma)/mitogen-activated protein kinase (MAPK)/ERK (Extracellular signal-Regulated Kinase) pathway with B-Raf inhibitors (dabrafenib, vemurafenib), or the endothelial nitric oxide synthase (eNOS) pathway (dasatinib, ponatinib) or the mammalian target of rapamycin (mTOR) pathway (everolimus, temsirolimus, ridaforolimus).

These drugs can be classified into two categories: small-molecule inhibitors that target the ATP-binding site of RTK intracellular domain (tyrosine kinase inhibitor, TKI), and monoclonal antibodies (mAbs) that either interfere with the RTK extracellular domain or target the VEGF ligand (anti-VEGF) [8,36] (Figure 1). 

VEGF inhibitors through antibody binding include bevacizumab, ranibizumab, aflibercept, and ramucirumab. Bevacizumab and ranibizumab are monoclonal antibodies (mAbs), and aflibercept is a recombinant fusion protein that acts as a soluble decoy receptor or VEGF trap. Ramucirumab is a fully humanized mAb that specifically inhibits VEGFR2. Among these angiogenesis inhibitors, some are used either alone or in combination with chemotherapy, while most TKIs are multi-kinase inhibitors targeting VEGFRs and other RTKs simultaneously [37] (Table 1).

### 3.2. Other VEGF Signaling Inhibitors

#### 3.2.1. RAF/MAPK/ERK Pathway

VEGF induces mitogen-activated protein kinase (MAPK) signaling pathways, especially ERK1/2 signaling. ERK activation is dependent on the upstream mediators Raf-1 and MEK (Mitogen-activated protein kinase) (Figure 1) [8]. Therapeutic inhibitors that target various portions of the MAPK/ERK pathway are currently in development as novel chemotherapies [99]. Some agents, such as vemurafenib and dabrafenib, specifically target B-Raf, an upstream component of the intracellular MAPK/ERK intracellular pathway. These agents may induce nephrotoxicity, particularly podocyte damage with foot process effacement. It has been shown in podocytes that B-Raf interacts with PLCε1 (Phospholipase C epsilon 1) and nephrin. Expression of these proteins is downregulated in kidney biopsies from patients treated with B-Raf inhibitors, leading to proteinuria due to slit diaphragm dysfunction [100,101]. Moreover, B-Raf inhibitors cause tubulointerstitial injury and electrolyte disorders (hypokalemia, hyponatremia, hypophosphatemia) [102].

#### 3.2.2. eNOS Pathway

Endothelial nitric oxide synthase (eNOS) is an essential molecule in mediating VEGF-induced angiogenesis and endothelial function [103] (Figure 1). It has been shown that dasatinib and ponatinib induce pulmonary arterial hypertension in patients, while ponatinib has been similarly associated with decreased NOS3 expression in vitro [104]. These anti-angiogenic drugs lead to an imbalance between vasodilator and vasoconstrictor agents, which results in cardiovascular toxic effects (hypertension, pulmonary hypertension, vascular events). Inhibition of eNOS reduces nitric oxide production and thus participates in vascular toxicities [105,106,107].

#### 3.2.3. mTOR Pathway

Another important downstream RTK target is mammalian target of rapamycin (mTOR) on the Pi3K (Phosphatidylinositol-3-kinase)/AKT signaling pathway (Figure 1). Inhibition of mTOR signaling reduces VEGF-dependent angiogenesis and endothelial cell proliferation [108]. It has been shown that mTOR inhibitors such as everolimus decrease VEGF levels and thereby block VEGF-induced angiogenesis. Temsirolimus, ridaforolimus, and everolimus have shown promising beneficial effects in the treatment of several cancers [109,110,111].

### 3.3. Current and Future Anti-Angiogenic Therapies

Over time, tumors acquire resistance against anti-angiogenic drugs and patients inevitably relapse. To improve patients’ survival, the combination of anti-angiogenic therapy with other agents such as kinase inhibitors, chemotherapy, DNA repair inhibitors, radiotherapy, and immunotherapy has been reported in the treatment of many tumor types [112]. VEGF also plays a key role in the regulation of the immune microenvironment by reducing endothelial T cell adhesion through deregulation of vascular cell adhesion molecule 1 (VCAM-1) and intercellular adhesion molecule 1 (ICAM-1) in endothelial cells. In addition, VEGF suppresses dendritic cell differentiation and T cell activation by increasing regulatory T cells [113,114]. Immune checkpoint inhibitors, such as antibodies against the programmed death protein 1 (PD-1), its ligand, programmed death ligand 1 (PD-L1), and the cytotoxic T lymphocyte-associated antigen 4 (CTLA-4), have been reported as effective in multiple cancers. Their combination with anti-angiogenic therapies increases the response rate and progression-free survival of patients [37,59,115].

## 4. Adverse Effects of Anti-Angiogenic Therapy

Given the implication of VEGF signaling in many physiological processes, targeted therapy leads to various side effects, notably renal complications, reported for many years now.

### 4.1. Hypertension

**Figure 2:**
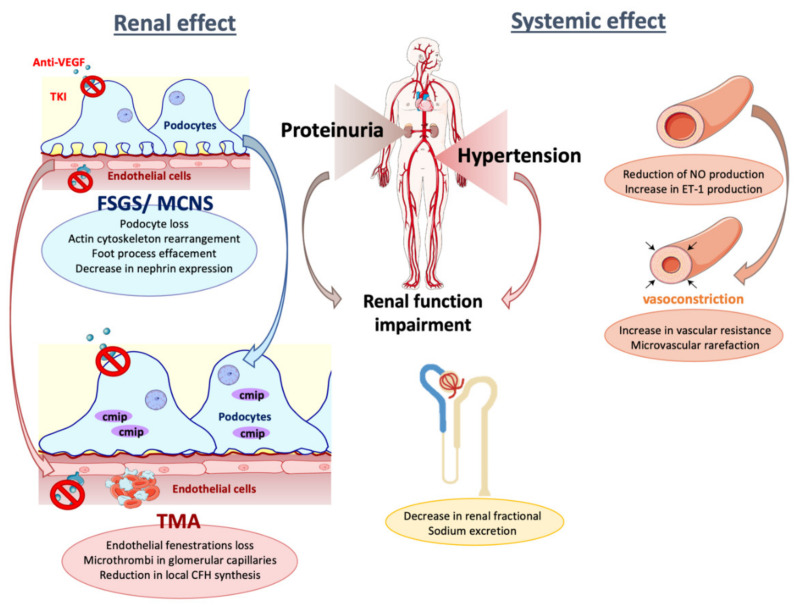
Adverse effects of anti-angiogenic therapy (TKI or anti-VEGF) on glomerular cells and vessels. CFH: Complement Factor H; CMIP: C-Maf-inducing protein; ET-1: endothelin-1; FSGS: focal segmental glomerulosclerosis; MCNS: minimal change nephropathy; NO: nitric oxide; TMA: thrombotic microangiopathy; TKI: tyrosine kinase inhibitors; VEGF: vascular endothelial growth factor.

Hypertension (HTN) is one of the most frequent adverse effects of VEGF inhibition and has even been associated with positive cancer outcomes [116,117]. The incidence of hypertension can vary, according to the anti-angiogenesis drug, between 4 and 87%, with an expected dose-dependent effect [49,118]. High blood pressure is considered as a class effect of TKI treatment, although the molecular processes underlying vascular toxicities induced by VEGF inhibitors still remain unclear. Several mechanisms have been suggested (Figure 2). First, it has been proposed that an increase in vascular tone is due to inhibition of VEGF-mediated vasodilation. VEGF stimulates endothelial NO synthase (eNOS) via phospholipase C-γ and phosphatidylinositol-3-kinase (Pi3K)-Akt pathways, promoting nitric oxide (NO) production to induce vasodilatation by smooth muscle cells [8,119]. eNOS inhibition by anti-angiogenic therapy increases vascular resistance due to the reduction in NO production. NO participates in the tubule-glomerular feedback, pressure natriuresis, and sodium balance [4,120,121]. Consequently, NO signaling inhibition may lead to the development of hypertension through sodium retention. Another mechanism implicated is the reduction in renal fractional sodium excretion (FENa), which is reduced in tubular segments by anti-angiogenic treatments, thereby contributing to volume-dependent hypertension [122,123]. Indeed, in tubular segments, sodium reabsorption occurs via the epithelial sodium channel (ENaC) and the sodium chloride cotransporter (NCC) in the distal convoluted tubule, connecting tubule, and collecting duct. Renal distal tubule and collecting duct epithelial cells express VEGFR2 [124]. In the study by Grisk et al., sunitinib-treated rats showed significantly reduced diuresis, natriuresis, and fractional sodium excretion, indicating that sunitinib stimulates renal sodium reabsorption [123]. However, Witte et al. showed that activated ENaC and NCC do not contribute to the pathogenesis of sunitinib-induced hypertension [122]. 

Other studies have also suggested a role for endothelin 1 (ET-1), a potent vasoconstrictor factor released by the endothelium. VEGF pathway inhibition by anti-angiogenic therapies induces ET-1 production in a dose-dependent fashion [125,126,127]. This results in hypertension and kidney dysfunction, such as in the case of pre-eclampsia [128]. ET-1 mediates vasoconstriction and cell proliferation by interacting with ETA (endothelin A) and ETB (endothelin B) receptors on vascular smooth muscle cells. Podocytes express the ETA receptor and constitute a target of ET-1 [129,130]. Indeed, it has been shown that ET-1 induces damage on glomerular podocytes and contributes to proteinuria [131].

eNOS and ET-1 pathways could increase reactive oxygen species’ (ROS) release. Oxidative stress may contribute to hypertension, potentially through ROS-mediated apoptosis of endothelial cells [132].

Another mechanism of endothelial dysfunction is microvascular rarefaction, which can be induced by anti-VEGF therapy. Indeed, VEGF is essential to maintain capillary network integrity. Hence, decreased capillary density may lead to increased systemic vascular resistance and pressure in larger vessels [133,134,135]. Patients with chronic kidney disease are more likely to develop hypertension after anti-VEGF treatment.

Hypertension occurrence under anti-angiogenic drugs may be influenced by genetic variants. It has been reported that single-nucleotide polymorphisms in *VEGF*, *VEGFR2, ABCB1 (ATP-binding cassette sub-family B member 1)*, and *eNOS* genes predict the rise in blood pressure and/or hypertension in TKI-treated patients [136,137,138]. Other genetic variants have been associated with bevacizumab-induced hypertension [139,140,141]. All these changes at the cellular level contribute to the increase in vascular tone and vascular remodeling during anti-VEGF treatment. The mechanisms causing hypertension under TKI appear to be more complex, also involving molecule-specific toxicities. 

### 4.2. Proteinuria

Proteinuria is the result of glomerular filtration barrier impairment in the glomeruli, releasing an abnormal amount of plasma proteins, mainly albumin, in urine. Proteinuria is a common side effect assignable to anti-angiogenic agents and a direct marker of therapy nephrotoxicity.

Incidence and rate of proteinuria are variable in different studies according to patient characteristics and the targeted therapies used [5,50,118]. The incidence of all-grade proteinuria is approximately 10–20% [5,50]. Despite this high frequency, most cases of proteinuria are asymptomatic or not severe, with nephrotic-range proteinuria (>3 g/day) occurring in 1–5% of patients depending on the duration of exposure to anti-VEGF therapy. However, patients with renal cell carcinoma have a higher incidence of nephrotic proteinuria, up to 7–8%, especially those having undergone nephrectomy [142,143].

On the renal biopsy from patients receiving anti-angiogenic drugs, the most common pathological findings are thrombotic microangiopathy (TMA), and focal segmental glomerulosclerosis (FSGS)/minimal change nephropathy (MCNS), with the latter related to podocyte injury. Inhibition of the VEGF-A/VEGFR2 signaling pathway seems to have differential downstream effects according to the therapeutic target. TKI treatment induces mainly MCNS and FSGS, whereas TMA was often observed in response to anti-VEGF [7,13,36].

The pathogenesis of proteinuria in patients receiving anti-angiogenic therapy likely relates to multiple pathways [13,144] (Figure 1). The two major cellular players involved in direct or indirect toxicity are glomerular endothelial cell and podocyte. Indeed, VEGF inhibition in glomerulus results in a loss of endothelial fenestrations, proliferation of glomerular endothelial cells (endotheliosis), and marked effacement of podocytes, which may lead to podocyte loss. In anti-VEGF-induced TMA, the glomeruli exhibit loss of fenestration, detachment of endothelial cells from the original basement membrane, and duplication of the glomerular basement membrane with interposition of cells between the two leaflets producing double contours. Other features include mesangiolysis and fibrin microthrombi in arterioles and glomerular capillaries. Characteristically, TMA in patients receiving bevacizumab appears to be limited to the kidney, with rarely systemic manifestations of TMA as seen in gemcitabine-induced TMA [145]. This confirms the essential role of VEGF in maintaining the integrity of the glomerular endothelial barrier [13,21,28,134]. Moreover, VEGF has a role in the coagulation cascade by inducing tissue factor (TF) expression in endothelial cells. TF induces thrombin formation from prothrombin, which, in turn, activates platelets and converts fibrinogen into fibrin to cause clot formation [146,147].

Complement activation can be observed in the glomerulus as a cause of TMA [148,149]. Complement factor H (CFH), considered as the most important inhibitor of the alternative complement pathway, prevents complement activation on the cell surface. Keir et al. have shown that VEGF regulates local inhibitory complement proteins in the kidney [150]. Accordingly, genetic deletion of podocyte *VEGF* inhibits local CFH synthesis. Moreover, anti-VEGF therapy may cause a reduction in local complement inhibitor synthesis and secretion, making cells more vulnerable to complement activation [150].

FSGS and MCNS are characterized by podocyte foot process effacement, which is the major structural correlate of nephrotic proteinuria. This change in podocyte shape results from actin cytoskeleton disorganization [151]. FSGS could be secondary to alterations of specific podocyte genes, such as *NPHS1,* encoding the transmembrane protein nephrin [152]. Nephrin is an important component of the slit diaphragm for glomerular filtration, and contributes, via its intracellular signaling, to maintaining the dynamic integrity of podocyte architecture by regulating actin cytoskeleton organization [20,152,153]. It has been shown that nephrin phosphorylation is essential to maintain slit diaphragm integrity [153,154], and compelling evidence suggests a cross-talk between VEGF and nephrin signaling pathways [17,20,155]. Indeed, nephrin interacts in vivo with VEGFR2 and activates downstream the Akt/PI3K pathway that regulates actin polymerization and stress fiber formation [17]. Conversely, VEGF signaling pathway inhibition leads to a decrease in *NPHS1* gene transcription. Moreover, administration of sunitinib in rats resulted in a dose-dependent decrease in nephrin mRNA levels [128]. Furthermore, an indirect toxicity effect on podocytes resulting from their cross-talk with endothelial cells exists. This was shown in an experience where conditioned medium from sunitinib-treated endothelial cells induced a decrease in nephrin expression [156].

Antagonism between the NF-kB transcription factor RelA (also known as p65) and the adaptor protein C-Maf-inducing protein (CMIP) seems to play a central role in this toxicity mechanism. Izzedine et al. showed that patients who developed TMA after anti-VEGF treatment displayed a high abundance of RelA in endothelial cells and podocyte nuclei, whereas CMIP was not detected [157]. However, glomerular lesions in biopsies from patients with MCNS or FSGS after TKI treatment were associated with increased CMIP expression in podocytes, while RelA was weakly detected [7] (Figure 1). The authors showed that RelA is a major negative regulator of CMIP transcription. As sorafenib inhibits RelA activation, it enhances CMIP expression in vivo and in vitro [7,30].

### 4.3. Kidney Dysfunction

Cancer patients are at risk for acute kidney injury, with a high prevalence of chronic kidney disease [158,159,160]. Kidney vulnerability to various nephrotoxic agents can be attributed to several functional properties. Kidneys are exposed to high levels of toxic drugs because they receive 25% of the cardiac output and process blood filtration, have a high tubular re-absorptive capacity (via specific transporters), and an ability to concentrate toxins within the medulla interstitium [161].

Subsets of patients receiving anti-angiogenic therapy and undergoing hypertension can also develop proteinuria and glomerular lesions (TMA, FSGS), leading to acute kidney injury, as these drugs may induce direct cellular toxicity on endothelial cells and/or podocytes.

Other mechanisms may be involved in this kidney dysfunction, notably the use of other systemic chemotherapies and the primary malignancy itself. Indeed, anti-angiogenic treatment can be used in combination with chemotherapy, which can induce, itself, renal toxicity. Bevacizumab, for example, has been approved in association with other chemotherapy drugs in non-small cell lung cancer (NSCLC), colorectal cancer, ovarian cancer, metastatic cervical cancer, and metastatic renal cell carcinoma (RCC) [35,112,159,162,163,164].

Surgical resection, which remains the gold-standard treatment for non-metastatic RCC, is also associated with risk of acute kidney injury [165,166]. The rate of chronic kidney disease (CKD) in patients who have undergone nephrectomy might be higher than that in the general population [166]. The nephron loss owing to nephrectomy can have a hypertrophic effect on the remaining nephrons, leading to glomerular hyperfiltration. Beyond a certain threshold of hypertrophy, increasing shear stress on podocytes promotes podocyte detachment and FSGS, which result in global glomerulosclerosis [167].

In addition, the frequent comorbidities present in cancer patients can have a potential impact on kidney function. Diabetes and hypertension are the main causes of chronic kidney disease and increase the risk of acute kidney injury [168]. Diabetes is a well-known condition associated with massive glomerular hyperfiltration [167]. Research concerning the long-term effects of anti-angiogenic therapies on kidneys is scarce. Patients who receive these treatments have an advanced cancer, many comorbidities, and kidney biopsy is rarely performed. Usually, but not always, kidney dysfunction resolves with dose reduction or drug discontinuation. It depends, in part, on the patient’s baseline renal function. However, these patients also present many risk factors for CKD (diabetes, older age, hypertension, and nephrectomy, for instance) that can lead to chronic kidney failure. Acute kidney injury and proteinuria induced by anti-angiogenic therapy may contribute to impairment of kidney function. To date, there is no predictive biomarker of kidney dysfunction for patients receiving these treatments. Creating a database registry for anti-angiogenic therapies and their renal adverse effects could be very helpful [169]. In the KDIGO (Kidney Disease Improving Global Outcomes) Controversies Conference on onco-nephrology, for patients with CKD, TKIs may be used at a lower-than-standard dose and increased secondarily according to individual tolerability and response [160].

### 4.4. Electrolyte Disorders

In addition to the nephrotoxic effects of anti-angiogenic agents, some drugs can cause damage to different tubular transporters. Electrolytic disorders encountered under TKIs are detailed in Table 1.

The proximal tubule expresses many transporters and receptors that are affected by pharmaceutical agents. For example, organic anion transporters (OATs) and organic cation transporter 2 (OCT2) are located on the basolateral side of proximal tubular epithelial cells, and multidrug and toxic compound extrusion (MATE) proteins are expressed on the apical side, with both ion transporters being involved in urinary excretion of creatinine [170,171]. For instance, vandetanib inhibits MATE1, MATE2, and OCT2 and induces electrolyte disturbances, such as hypocalcemia, hypokalemia, hyponatremia, and decreases in creatinine clearance [74,172,173]. Sorafenib frequently causes hypophosphatemia, possibly linked to low vitamin D levels in serum, but the underlying mechanism remains unclear [51,52,174]. One study suggested a fibroblast growth factor 23-independent mechanism and showed a decrease in urinary phosphate and calcium levels over time with a decreasing trend for FGF23 (fibroblast growth factor 23) levels. In this work, a decline in serum C-telopeptide of type I collagen levels was also observed, suggesting that sorafenib inhibits bone resorption through inhibition of osteoclast activity [51]. Hypophosphatemia appears to be a common adverse effect of multitargeted TKIs, observed among patients treated with sorafenib, lenvatinib, and regorafenib.

Hyponatremia is also frequently encountered in patients on TKIs, especially under sorafenib [53]. The underlying mechanism could be a syndrome of inappropriate secretion of antidiuretic hormone, but it should be pointed out that this effect can be paraneoplastic and developed independently of any treatment [94]. Digestive disorders, especially diarrhea, are frequent under TKIs and can also lead to hyponatremia.

This reflects a completely divergent damage pattern of anti-angiogenic agents, even though they target the same VEGFR pathway [145].

### 4.5. Cell Damages

#### 4.5.1. Cytoskeleton and Focal Adhesion Architecture

Dasatinib, a TKI used in chronic myeloid leukemia, induces nephrotoxicity through altered podocyte actin cytoskeleton, leading to injurious cellular biomechanics. Podocytes exposed to dasatinib show significant changes in focal adhesions, actin cytoskeleton, and cell morphology [6]. To date, only two studies have shown a direct link between TKIs (sorafenib, dasatinib) and an impairment of podocyte cytoskeleton [6,7].

#### 4.5.2. Tubular Cell Apoptosis

In a study using HK-2 cells (human tubular epithelial cells), sunitinib induced renal toxicity in a dose- and time-dependent manner by inducing apoptosis [175].

#### 4.5.3. Autophagy and mTOR Signaling

Autophagy is a metabolic process helping cells to maintain homeostasis via recycling the aging or damaged organelles. Anti-angiogenic agents reduce tumor microvascular density and increase tumor hypoxia and, therefore, upregulate autophagy activation of tumor cells to maintain cell survival and normal metabolism. This increased autophagy is considered to play a cytoprotective role in most cases. However, the outcome of autophagy under certain conditions may be the opposite, promoting cell death, though the mechanism of this condition-dependent role is still unclear.

If autophagy can constitute a cytoprotective mechanism [176], it can also exert cytotoxic effects by causing, in some cases, “autophagic cell death” in several cell types [177,178,179].

It is known that autophagy is an essential process for the podocyte to prevent senescence [180]. In the kidney, several studies have outlined the importance of mTOR signaling in the maintenance of podocyte integrity, primarily through regulation of autophagy, as deletion of either of one of the two mTOR functional complexes resulted in glomerulosclerosis [181]. In a murine model with podocyte-specific deletion of mTOR, mice developed proteinuria and even end stage renal disease (ESRD) by the age of 5 weeks, with a disrupted autophagic flux in podocytes [182].

In diabetic mice, through specific endothelial cell knockdown of *atg5*, a key gene for autophagic vesicle formation, we observed increased glomerular endothelial lesions. This suggests that mTOR signaling pathway is also important for endothelial cells [183].

Thus, it can be hypothesized that inhibition of VEGF signaling, thereby inhibiting the mTOR signaling pathway through PI3K/AKT, could lead to disrupting autophagy, ultimately resulting in cell death and particularly in podocyte loss. This could explain the proteinuria observed in patients treated with anti-angiogenic drugs.

## 5. Conclusions

It is now well established that anti-angiogenic therapies induce kidney toxicity, such as hypertension, proteinuria, and kidney dysfunction. The best management strategies to counteract such toxicity are largely unknown. Renal monitoring is crucial for patients who receive these treatments, both before initiation and also during therapy, to identify renal adverse events [160]. French recommendations for good practice have been published, emphasizing the need for multidisciplinary coordination in the follow-up of these patients [184].

Currently, there are no therapies to treat these renal complications, apart from drug discontinuation, dose reduction, or symptomatic treatment with angiotensin-converting enzyme inhibitor or angiotensin receptor blockers due to their renoprotective effect. It is essential to better understand the underlying mechanisms to reduce nephrotoxicity without inhibiting the anti-angiogenic effects on tumor.

## Figures and Tables

**Table 1:** Incidence of renal manifestations and electrolytic disorders under anti-angiogenic targeted therapies.

Drugs	Molecular Targets	Tumor Targets	Adverse Events (Incidence)	Electrolytic Disorders	References
**Monoclonal antibodies**					
**Bevacizumab**	VEGF	CRC, NSCLC, RCC, GBM, epithelial ovarian cancer, primary peritoneal cancer, cervical cancer, fallopian cancer, glioblastoma, ocular diseases	HTN (23–41%), Proteinuria (2–32%)	Hypophosphatemia, Hyponatremia	[38,39,40,41,42,43,44,45,46,47,48]
**Ranibizumab**	VEGF	Ocular diseases	HTN, Proteinuria	-	[38,39]
**Ramucirumab**	VEGFR2	CRC, NSCLC, GC	HTN, Proteinuria	-	[38,39]
**Recombinant fusion protein**					
**Aflibercept**	VEGF	CRC, ocular diseases	HTN, Proteinuria	-	[38,39]
**Multitargeted TKI**					
**Sorafenib**	VEGFRs, PDGFRs, RAF, c-Kit, FLT3, Ret	RCC, HCC, DTC	HTN (17–55%), Proteinuria (10%)	Hypophosphataemia (16–85%), Hyponatremia (39%)	[49,50,51,52,53,54,55,56,57,58]
**Sunitinib**	VEGFRs, PDGFRs, FLT3, CSF1R, Ret	RCC, GIST, pNETs	HTN (22–60%), Proteinuria (10–65%)	Hypophosphatemia, Hyponatremia	[59,60,61,62,63,64,65,66]
**Pazopanib**	VEGFRs, PDGFRs, FGFR1, c-Kit	RCC, STS	HTN (40–52%), Proteinuria (13.5–18%)	Hypophosphatemia (34%), Hypocalcemia (33%), Hyponatremia (31%), Hypomagnesemia (11%)	[49,50,67,68,69,70,71,72,73]
**Vandetanib**	VEGFRs, EGFR, Ret	MTC	HTN (23.5–84%), Proteinuria (5.6–26%)	Hypomagnesemia (10–40%)Hypocalcemia (4–29%)Hypokaliemia (4–17%)	[5,50,74,75,76,77,78,79]
**Axitinib**	VEGFRs, PDGFRs, c-Kit	RCC	HTN (40–64%), Proteinuria (4.6–23%)	Hyponatremia, Hypophosphatemia (13%), Hypocalcemia (39%)	[5,49,50,80,81,82,83]
**Regorafenib**	VEGFRs, PDGFRs, FGFRs, Tie2, c-Kit, Ret, RAF	GIST, CRC, HCC	HTN (13–59%), Proteinuria (7–9.4%)	Hypophosphataemia (5–18%)	[5,49,50,55,84,85,86]
**Cabozantib**	VEGFRs, c-Met, AXL, c-Kit, FLT3, Ret	MTC, RCC	HTN (7–16), Proteinuria (6%)	Hypophosphatemia (4–8%)	[5,87,88,89,90,91]
**Nintedanib**	VEGFRs, PDGFRs, FGFRs, SRC	IPF, NSCLC	HTN, Proteinuria	-	[92]
**Lenvatinib**	VEGFRs, FGFRs, PDGFRa, Ret, c-Kit	DTC, RCC, HCC	HTN (45–100%), Proteinuria (26.9–100%)	Hypophosphatemia (45%)	[5,55,93]
**Dasatinib**	BCR-ABL, SRC, LCK, YES, FYN, c-Kit, VEGFR, PDGFR	CML, Ph+ ALL	Proteinuria	Hyponatremia	[94,95]
**Ponatinib**	VEGFRs, BCR-ABL, FLT3, Ret, c-Kit, FGFRs, PDGFR	CML, Ph+ ALL	HTN (9–32%)	-	[96,97,98]

CML: chronic myeloid leukemia; CRC: colorectal cancer; CSF1R: colony stimulating factor 1 receptor; DTC: differentiated thyroid cancer; FGFR: fibroblast growth factor receptor; FLT3: fms like tyrosine kinase 3; GBM: glioblastoma multiforme; GC: gastric cancer (or gastroesophageal junction adenocarcinoma); GIST: gastrointestinal stromal tumor; HCC: hepatocellular carcinoma; HTN: hypertension; IPF: idiopathic pulmonary fibrosis; LCK: lymphocyte-specific protein tyrosine kinase; MTC: medullary thyroid cancer; NSCLC: non-small cell lung cancer; PDGFR: platelet-derived growth factor; Ph+ ALL: Philadelphia-chromosome-positive acute lymphoblastic leukemia; pNETs: progressive pancreatic neuroendocrine tumors; RAF: rapidly accelerated fibrosarcoma; RCC: renal cell carcinoma; STS: soft tissue sarcoma; VEGF: vascular endothelial growth factor; VEGFR: vascular endothelial growth factor receptor.

## Data Availability

Data sharing not applicable. No new data were created or analyzed in this study. Data sharing is not applicable to this article.
